# Dr. P. J. Bauduy’s Cases of Tetanus Successfully Treated

**Published:** 1842-07-02

**Authors:** 


MEDICAL EXAMINER.

NEW SERIES.

No. 27.] PHILADELPHIA, JULY 2, 1842. [Vol. I.

### *Cases of Tetanus successfully treated.*

To the Editors of the Medical Examiner.

Gentlemen,—The happy termination of so formidable a disease as Tetanus is so rare, that I am induced to offer you the following cases for publication. They occurred within the last four years in the practice of a friend (the late Dr. P. J. Bauduy,) in the Island of Cuba. They are the rough notes taken at the bedside, and were not intended for the public eye in their present form being subject to future revision. This intention their author was not permitted to fulfil, falling a victim to the climate and his own exertions last autumn; and I have not considered myself justifiable in making the slightest alteration.

With sincere regard, your friend,

M. Clymer.

*Philadelphia*, June 29th, 1842.

### *Case of Traumatic Tetanus.*

Manuel de Alma, ætatis 25, a stout, strongly constituted young man, a labourer, and very healthy, about twenty days ago, while propping up a post, he fell in the hole which had been dug to receive it, and a small splinter three-quarters of an inch long, and as thick as a pin, was driven under the integuments of his left thigh, between the Sartorius and triceps adductor muscles. A few days afterwards he felt slight pain in the wound, and found that a small quantity of pus had collected about the splinter. He gave vent to it, but still did not extract the extraneous body. About eight days after he began to feel much uneasiness about the jaws, and this rapidly increased to rigidity and difficulty in opening them freely. He was now advised by his friends to cease working, and consult a physician; but, regarding their fears as foolish, he continued at hard work for several days more, until last Sunday, the 21st October, fifteen days after the injury, he got on his horse and rode to the neighbouring village to consult a physician. Upon arriving there he was suddenly seized with a strong convulsive spasm of the whole body, and fell from his horse. He had to be carried home, and put to bed, as the spasms continued at short intervals.

Oct. 23d. His friends finding that he was growing much worse, sent for me to consult with his attending physician. The following is the state he was in:—No fever; pulse natural; skin rather more hot and dry than natural; great rigidity of jaws, cannot open them more than an inch; great anxiety of countenance; rigidity about all the muscles, moving the jaws and larynx; great difficulty in swallowing; his bowels have not been opened since three or four days; can pass his urine only with the greatest difficulty; great rigidity of limbs; no opisthotonos visible; every two or three hours he has the shooting pain from præcordial region to spine (so characteristic of this disease,) accompanied by strong spasms of all the muscles of the trunk, the extremities not being involved. Belly hard and tense; dull sound on percussion, except over the sigmoid flexure of colon, where there is some tympanitis; recti muscles contracted, rigid, and very hard. His physician is treating him with antispasmodics, chiefly musk and assafœtida. I proposed a more active treatment, but as we could not agree, I thought it best to withdraw.

24th. To-day the friends of the patient, as well as himself, being dissatisfied with the attending physician, and finding that the disease was fast advancing towards a fatal termination, dismissed him, and sent for me to take charge of the case. This I at first refused to do, through motives of delicacy towards a fellow practitioner, but was overruled by the strong desires and entreaties of the patient and his friends. Saw him then at five P. M., of this day. Found all the bad symptoms increased; can no longer pass his urine; can scarcely open the jaws at all; spasms stronger and more frequent; has had no stool; considerable anxiety of countenance; can swallow, but with the greatest difficulty, and suffers excruciating pain. I immediately dilated the wound freely, (the splinter has been extracted only a few days before by his physician,) and applied to it *the actual cautery*, and ordered hot emollient cataplasms to be kept over it. An injection of oil of sweet almonds, Castile soap and molasses, in which a clove or two of garlic should be bruised, to be given immediately, and repeated every four hours until the morning. Frictions of hot oil, with bruised garlic, over abdomen and throat, and cataplasms of tobacco over pubal region, in order to overcome the spasm of sphincter of bladder. Ordered him for next morning a pill of ten grains of the sub-nitrate of mercury—a medicine of much power much used in this country, to which I believe its use is almost entirely confined; it is powerfully emetic, cathartic and diaphoretic; its effects in traumatic tetanus are often wonderful, and, altogether, it is a medicine possessed of such important powers as to merit much attention. It is an empirical remedy, but one that deserves to be tried by the profession. I propose, on some other occasion, to furnish the readers of your Journal with an account of its powers, and the diseases in which I have found it useful. Ordered likewise frictions of hot oil, in which should be dissolved nitrate of mercury, one grain to the ounce, to the abdomen, thighs, knees, neck, jaws, and inside of shoulders, to be repeated every three or four hours. Luke-warm water, with a few drops Spt. Æth. Nitrosi, for drink.

25th. Saw him about 1 P. M. The nitrate of mercury produced copious vomiting and four or five large fœcal evacuations; has passed his urine without much difficulty, and in large quantities; is perspiring pretty freely; belly much softer; says he feels much better; has had no shooting pain from sternum to spine; looks much more cheerful; pulse natural; slight thirst. Ordered a small blister to be put on the wound. Ordered two or three purgative injections, to be given in the course of the afternoon.

Eight P. M. Saw him again. Injections produced two copious stools. Gave him half a grain sulph. morphia. Ordered frictions of nitrate of mercury to be continued, and ten more grains of the same to be taken early next morning.

26th. Eight A. M. Slept well last night, but suffered from slight difficulty in urinating. Took the nitrate of mercury at 5 A. M. Has vomited but once, and has not been purged; belly a little harder; has had slight returns of the shooting pain from sternum to spine; is bathed in perspiration; is more dejected—says he will die. Ordered a strong purgative enema every hour till he evacuates freely, and frictions of nitrate of mercury to be continued.

Six P. M. Profuse perspiration still continues; complains of tongue; lips and whole mouth paining him; pulse 65, soft, rather irregular; belly much softer; has urinated freely, and had several large evacuations; opens his jaws nearly as well as in health; tongue coated with white fur, edges and tip red. Ordered another purgative injection; frictions still to be continued; tobacco cataplasms to abdomen; sulph. morphiæ gr. ss., q. q. h.

27th. Five P. M. Passed a good night. Had several stools, and complains of tenesmus, and passes blood in the evacuations; urinates very freely, but has still very slight pain; belly quite as soft as natural; has had but very few and very slight shooting pains from sternum to spine; opens jaws freely; swallows easily; cheerful; much thirst; pulse 84, soft, regular; tongue coated with thick white fur; complains much of mouth and throat; craves for food; blister on wound has had no effect but that of causing abundant suppuration. Sulph. morphiæ half a grain, to be continned quaque q. hora; frictions to be still continued, and tobacco cataplasms. As I think that there is now slight opisthotonos, I ordered strong tartar emetic ointment to be rubbed in between shoulders. Injections of starch, with half a grain of sulph. morphiæ q. 6 h., if evacuations continued. Basilicon to the wound. Weather since yesterday morning very unfavourable—cold north-easterly wind, with continual showers of rain.

29th. Could not see him yesterday on account of continued heavy rain. To-day, at 10 A. M., found him better; no shooting pains; jaws open quite freely; no opisthotonos, but belly is harder, and there is considerable difficulty in urinating; bloody stools were stopt by the injections on the night of 27th; profuse salivation; mouth, gums, and lips very much swelled, full of small ulcers; tongue covered with thick fur, edges very red, and small ulcers on them; slept very well last night; not much thirst or appetite; pulse 72, soft, regular. Ordered frictions of nitrate of mercury to be stopt; ℥i. of castor oil to be given, and hot tisan of elder blossoms and borage leaves, with sweet spt. of nitre, for drink. Frictions of hot olive oil, spts. ammoniæ, and tinct. opii over jaws and throat. Frictions of tartar emetic to be discontinued, as pimples are very numerous and sore. Gargles of lead water and infusion of rose leaves. Sulph. morphiæ gr. ss.. q. 6 h.

31st. Ptyalism still continues, but less; mouth less swelled, less painful; tongue coated with thick white fur, resembling coat of white lead paint, blackened in many places by gargle; jaws open naturally; belly still hard, much difficulty in passing the urine; bowels regular; no thirst or appetite; chest very prominent, and some rigidity of muscles of back; no shooting pains; no opisthotonos; pulse 72, regular. Ordered a few drops tinct. valerian to be added to his tisan; sulph. morphiæ, quarter of a grain; camphor three grains, every three hours. Emollient fomentations over pubal region; frictions of oil, and spts. of ammonia, &c., to be continued to fauces; wound suppurates freely; gargle continued, &c.

Nov. 2d. Five P. M. Is better; pulse 72, regular; quite cheerful; opens jaws very well; less prominence of chest; less rigidity of muscles of back; ptyalism nearly ceased; mouth much better; gums less swelled and less spongy; tongue still covered with same white thick fur, cleansing on edges and tip; bowels very costive, and great difficulty in passing his urine—amounts to almost total retention; valerian was not given; occasionally he has slight shooting pain from sternum to spine, when belly becomes very hard, limbs flexed and rigid; belly otherwise less hard; quite cheerful; sleeps well; appetite improved, and slight thirst. Ordered purgative injections to be repeated until he evacuates freely; root of valerian to be added to tisan. Introduced catheter, found considerable resistance at neck of bladder, but by holding it there some time, and pressing it steadily but very gently forward, and at the same time diverting patient's attention, I finally overcame the spasm; drew off but a small quantity. Ordered for next morning compound senna tea; a little more nutritious aliment allowed; tisan and other medicines continued as before, except morphia, which is to be suspended.

5th. Much better; no pain whatever; purge had operated very freely; his bowels still continue loose. Ptyalism has increased. Tongue clear, except at back part, where there is still a thick fur. Gums very red; tender, but hard. Pulse 72, natural; belly soft; appetite very good; sleeps well; walked about the room to-day; no longer any difficulty in passing the urine; jaws open freely. Ordered gargle of Tinct. Myrrhæ, Tinct. Guaiac., and Infus. Cinchonæ.

8th. Much better; convalescent. Ordered all medicine except gargle to be stopt.

12th. Met him riding out; is quite well; wound closed.

### *Case of Idiopathic Tetanus.*

Francisco de la Torre, ætat. 3, has always been a strong hearty boy. Nine days after birth was seized with rigidity of muscles that move the jaws, which daily increasing for several days, he could not open his mouth; could not suck; and it was with the greatest difficulty a small quill could be forced between the jaws so as to feed him. He had at same time much fever; was constipated; had copious sweats, and broke out over whole body with an eruption, (probably lichen;) no other symptoms can be remembered by mother. He was cured by applying a magnet stone to his jaws at the end of twenty-one days!!!! He was perfectly well till within two months ago, when, having symptoms of worms, his mother gave him some dolichos pruriens, which caused him to pass more than forty lumbrici. His appetite improved, and he was perfectly well until about two weeks ago; having slept under an open window, he woke next morning with a slight contraction of muscles of face, giving him a singular expression of countenance. This continued until yesterday, without any other symptom but *obstinate constipation.* His parents were not alarmed, thinking it was only that partial paralysis of a few muscles from cold, so often met with in this country. (They had tried their favourite magnet stone without any effect.) But yesterday he was seized with fever, which not abating, they sent to-day for me.

September 4th. Pulse 130, full, quick. Skin dry, hot. Tongue slightly furred; rigidity of jaws; cannot be opened quite an inch. Abdomen hard, tumid, but not tympanitic. Has not had a stool for more than five days; eyes half closed; livid hue round the lids; slight opisthotonos. Chest very prominent; decubitus dorsal: upon being disturbed starts up in a violent spasm, resting upon his hands, back of head, and the heels. Pulse much accelerated during the spasm; much thirst; urinates freely; designates forehead as seat of pain. Ordered immediately Ol. Ricini, ℥i., with forty drops Spt. Turpentine; to be followed in two hours with an enema of Ol. Ricini, ℥iss., and Spt. Terebinth, 100 drops; for drink milk and water, with a few drops Sweet Spirits of Nitre; boiled milk for food; and a warm aromatic bath every six hours.

At 10, P. M., found no change; had had two stools; semi-fluid, and had perspired freely after the baths. Ordered a purgative injection, and the following powder every three or four hours. Calomel gr. i., Pulv. Opii. one-fourth of a grain, and Pulv. Ipecac. one-sixth of a grain. Also friction of hot oil and garlic to abdomen and chest. Cataplasms of tobacco leaves to jaws and belly should rigidity increase.

5th, 8 A. M. Has more fever; pulse 140, increases during spasms, which are much more severe, but only take place when he is disturbed. Skin hot but moist; had a stool last night without having taken the enema; intense thirst; belly hard; chest more prominent; opisthotonos rather increased. Complains much of his head; eyes nearly closed; jaws the same; asks for food. Ordered warm baths and powders to be continued. Evaporating lotions to head; blister to nucha; purgative injection every six hours; acidulated drink with Spt. Æth. Nitre; food, milk and toasted bread.

At 8 P. M. Pulse 150; skin cooler; moist; has had no evacuation from the enemas; opisthotonos diminished; tongue cleaner; opens jaws rather more; spasms as usual, only when he is disturbed; still great thirst; belly tumid, hard, and now tympanitic. ℞. Ol. Tiglii. gtt. ij. Ol. Ricini, ℥j. M. ʒi. q. 6 h. until he evacuates. Frictions to be continued, as well as warm bath. Powders to be continued also. Twelve drops Tinct. Opii. at bed time.

6th, 9 A. M. Has had two stools, having taken two doses of the Croton oil mixture. Pulse 120, soft; skin dry, but cool; expression of countenance better; jaws open much more freely; belly softer, not tumid or tympanitic; opisthotonos much diminished; tongue covered with slight white fur; more lively; plays on the bed; talkative; still thirsty, but less so; but little appetite; less pain in the head; blister not risen well as yet. Ordered to be dressed, when well risen, with basalicon, and half a grain Sulph. Morphiæ q. 6 h. Warm bath to be continued. Powder q. 3 h. Croton oil at 6 o'clock, should he not have an evacuation, and repeat q. 6 h. until it produces the desired effect. Frictions continued.

7th, 8 A. M. Pulse 112. Has had but one evacuation, hard fæcal matter; has taken three doses of Croton oil mixture; complains of much headache; remains very quiet and dull; looks as if affected by opium; belly hard, but not tumid or tympanitic; jaws much more rigid; no opisthotonos; skin cool, dry; no thirst or appetite; spit a little blood this morning just after a spasm. Suspecting that he might have more lumbrici in alimentary canal, ordered a small quantity of Dolichos Pruriens, to be followed six hours after by ℥ss. of Ol. Ricini, with twenty drops Spt. Turpentine, to be repeated every four hours until it operates. Continue powders. Discontinue Morphia on blister.

N. B. Weather, which had been very fine, changed last night; is now very moist. Wind very high; cold and moist.

7 P. M. Found him bathed in perspiration, and sleeping soundly; had just taken the bath, and had a warm friction over whole body of hot oil. Pulse 96; skin hot; expression of face good; abdomen soft, not tympanitic; had but one stool, that about an hour ago, and produced by an enema; has had no appetite; has been very cross; no thirst. Ordered him not to be disturbed; but when he should awake, ʒii. of Croton oil mixture to be given, and powders continued.

8th, 9 A. M. Has had four stools, very yellow colour; took the ℥; of Croton oil mixture; opens jaws better than yesterday, but still are quite rigid; belly rather hard, but not tympanitic; recti muscles very hard; no longer complains of pain in the head. Pulse 120, soft, full; skin dry, cool; very thirsty; no appetite; slept well; no opisthotonos; blister does not suppurate. Weather very rainy and cold, and very high wind. He is in a room that is much exposed. Ordered blister dressed with savine cerate, and half a grain of Morphia. Ten drops Tinct. Opii. every six hours. Powders, warm bath, frictions to be continued.

9th. Could not see him last night, as weather was very rainy and stormy. Had two stools; took Croton oil mixture twice; slept well; passed a very large live lumbricus this morning; much appetite; much thirst; complains of mouth; has large apthæ in it; does not complain as before; playful; belly hard, but not tumid; expression of eyes improved; the motions of head, which from first day had been very stiff, are now quite free; opens jaws well; no opisthotonos. Treatment continued as before. Dolichos Pruriens to be repeated to-morrow morning.

10th. Six P. M. Had three stools since yesterday morning, without taking the croton oil mixture; gay and playful; good appetite; did not sleep well last night; jaws open freely and can protrude the tongue, which he had not been able to do before; tongue covered with a light whitish fur, and several aphthæ; no opisthotonos; belly less hard; asks for drink frequently; pulse 125, soft; has no headache; treatment continued. As blister is almost dry, tartar emetic ointment, ℈ij. to ℥j. of lard to be rubbed upon spine every three or four hours.

11th. Six P. M.—Has just come out of warm bath; is in a profuse perspiration; skin hot; pulse 125; had two stools to-day after taking croton oil mixture once; opens jaws as well as in health; complains much of aphthæ; spits occasionally a little frothy blood, probably from throat; complains much of pain about lower jaw, submaxillary glands, &c.; otherwise seems perfectly well; tartar emetic pustules beginning to appear. Treatment continued.

12th. Six P. M.—Find all symptoms of disease disappeared; stopt all medicine, and ordered only the baths and frictions to be continued, with one-eighth of a grain of sulph. morphiæ at night. Weather has been very fine again the three last days.

15th.—Called to see him; find him playing about the house perfectly well.

Nov. 25th.—Saw him to-day; is very hearty and gay; slight difficulty in articulating has remained.

*Case of Numerous Incised Wounds, attended with great and dangerous Hæmorrhage, &c.*

On the 15th of March, 1839, an insurrection broke out among the negroes, at a neighbouring sugar estate. Two men were killed, and several dangerously wounded by the insurgents. Among several cases that I attended, I select the following as one evincing how far a good constitution will go towards effecting a recovery from injuries that in most individuals would prove fatal, and the power which some individuals have of resisting hæmorrhages that would seem necessarily mortal. The subject of this case is a negro, the slave of a neighboring gentleman, who, hearing the shrieks attending the outbreak of the insurrection, came over to the spot with the slave he considered as most faithful to him. On their arriving at the scene of this dreadful murder, the negroes rushed on them, and the faithful slave placed himself before his master, so as to ward off the blows aimed against him. He grappled with the foremost, and threw him to the ground, falling himself over his antagonist, and in this position was literally hacked to pieces by the infuriated insurgents. This was at 5 A. M. I arrived at about 8 A. M., and immmediately proceeded to dress the wounds of several white men, and at about 11 A. M., having got through them, proceeded to examine what I had taken for the corpse of one of the revolted negroes, which I saw lying in the corner of the room. My astonishment was great to find that there was still life in the body, and that it was the faithful servant I had heard spoken of, but whom I thought dead. He was speechless, cold as ice, almost literally floating in a pool of his blood, and all his clothes saturated with same. Could feel no pulse at wrist, but heart beating faintly. Finding that I should be obliged to amputate his arm, I sent off for my case, and in the meanwhile proceeded to dress his wounds, which were as follows: On the left arm, a sabre cut had divided the four fingers of the left hand at about the articulation of the first and second phalanges, leaving their extremities hanging only by a few shreds of tegument. Another had penetrated the dorsum of the hand, fracturing the metacarpal bones of the four fingers, and cutting down to the palmar aponeurosis. A third had laid open the whole articulation of the wrist, with the fore-arm, leaving the hand adherent to the fore-arm merely by a few shreds of the palmar teguments, and the divided ulnar and radial arteries gaping out, but not bleeding. A fourth cut had divided the muscles on the back of the fore-arm at about the upper part of its lower third, and fractured both the radius and ulnar,—A fifth sword cut had divided the muscles of the lower and internal front of fore-arm at about its middle part, cutting through the ulna down to the radius. On his right arm he received the following wounds: Thumb split down by a sabre cut from its extremity to articulation of its phalanges, with fracture of the bone of the second phalanx. A second on the front part of fore-arm at lower third, dividing merely the teguments. A third, at the lower part of the middle third of the front of fore-arm, dividing the muscles down to the flexor sublimis digitorum; and a fourth on the internal part of the back of fore-arm, at about the lower part of its upper third, fracturing the ulna, and leaving its upper extremity projecting out slightly from the wound. On the head, neck and face, he had received numerous wounds; one, about four inches long on the left side of the neck, divided the integuments from the mastoid process to near the lower part of the larynx, cutting down to the sterno-mastoid muscle, and probably divided the external jugular; but as it was filled up by a pretty firm coagulum, I did not like to risk probing and poking at it in search of this vessel. Another sabre blow sliced off the whole left cheek, beginning a fourth of an inch below orbitar margin of submaxillary bone, and cutting down to near the alveolar processes, leaving the cheek hanging by a small strip, three-fourths of an inch broad. Another large wound on the left temple had penetrated to the bone, and divided the anterior temporal artery, and ten more sabre cuts on the scalp in every direction, all cutting down to the bone, and two of them, one answering pretty nearly to coronal suture at the superior posterior part of frontal bone, and the other dividing this at right angles just below sagital suture on left parietal bone, penetrated through the exterior plate of the bones. Several of the others chipped off small pieces of the bones, for as his skull seems remarkably thick, the swords mostly glanced off of it.

When first placed on the operating table, he did not bleed from a single wound, but there was a slight oozing of serous fluid from several of the larger ones. I have already said that he was cold—almost speechless; he whispered to me for water, and seemed parched with thirst. I gave him, with a view of rousing up the vital powers, which seemed almost extinguished, Liq. Ammon. [Aromat.?] ℥ss. with Tinct. Opii ʒi. and strong brandy ℥iv. in half a tumbler of water, and proceeded to dress his wounds. After shaving all the hair off the scalp, I washed his wounds to clear them of the earth and hair that they contained, and stitched them as neatly as possible with the interrupted suture, but not uniting the lips closely, for fear, if much inflammation and swelling should take place, that the sutures might by their irritation produce an erysipelas, that in the debilitated state which the patient must remain in would most certainly have proved fatal. I then covered them with lint, moistened with a simple dressing of equal parts of brown sugar, olive oil, and claret wine boiled up together—(Samaritan balsam)—the same was done by the wound of the neck, except that I did not remove the coagulum in it; and beside the sutures I placed several strips of adhesive plaster over it; and over all a thick compress fastened by a bandage placed round the neck, but so as not to produce any pressure on the large vessels at the side of the neck. The flap that had been sliced away from the superior maxillary and molar bones of the left side of face, leaving them bare, now hung down perfectly cold, and the muscles were of a bluish or rather livid hue. I, however, determined to make an effort at producing re-union. I washed it clean, and tacked it with four interrupted sutures neatly in its place, covered it with lint, and then with a thick compress, which I fastened in its place by adhesive straps. I dressed the wounds of right fore arm with adhesive straps, reduced the fracture of the ulna, and passed a bandage up loosely from the fingers to the elbow, placed a compress over the upper extremity of the fractured bone, and placed on the arm two pasteboard splints, well soaked previously, which I fastened loosely, one on the back and the other on the front part of the fore arm. I now felt his pulse, and found that it had risen considerably, being as strong as I could expect under the existing circumstances; the heat of the body had also in a great measure returned, and he now spoke plainly, complaining of much thirst; the heart beat much stronger and more regularly; I therefore decided that sufficient reaction had taken place, and amputated his left arm just below the elbow joint. He lost more blood during the operation than I well liked, through the clumsy management of my assistant, who had charge of the tourniquet. After dressing the stump, I administered sixty drops more of laudanum, and put him in a comfortable bed, wrapping him up warmly. Ordered acidulated gum water for drink, and two table-spoonfuls of broth every hour, as he had lost so much blood, and been so long without nourishment of any kind. It was 2 P. M. when I finished the amputation and put him to bed; he had received his wounds at about 5 A. M.: he had been all this time without any dressing whatever on his wounds, and what prevented him from bleeding to death is to me a mystery—probably it was syncope.

16th. Saw him at 8 A. M. He has no fever, but much thirst; pulse stronger; asks for food; did not sleep last night. Ordered a few drops of Spt. Æth. Nitros, added to drink; arrow root and rice, and a few table spoonsful of broth, to be given frequently.

17th. Strong fever; wounds are all suppurating copiously; dressed them all. Ordered laxative enemas. He continued now doing better and better every day.

April 1st. Was sent for to check a sudden hæmorrhage from the arm, which I found owing to a ligature (the ulnar artery) having come away; checked it by compresses wet with lead water, and appropriate bandaging. This ligature had been put on by my assistant, and I remarked to him at the time that he took up too much of the cellular tissue, and that I feared it might give me trouble. At this date all the wounds of head, neck, and face, with the exception of the two that involved both plates of cranial bones, were healed, almost all by the first intention. On the fourth day, observing much œdema of the cheek on the left side, and that the eye was much inflamed, with its lower lid very œdematous, I cut away the sutures that confined the flap of the cheek just below the eye, and next day observed that it had answered the purpose intended, as the inflammation and œdema had subsided. The wounds of the right arm are healed, with the exception of that answering to the fracture of the ulna, which is, however, fast filling up with very kind granulations. Amputated arm doing very well.

15th. To-day all his wounds are healed, and finding that no solid union has taken place between two fragments of ulnar, I applied the appareil immobile to the fore-arm.

17th. As he complains much of the arm since the application of the immoveable bandage, I took it off, and found that it had caused the cicatrix of the wound answering to fracture, and the one immediately below, to slough off; I was, therefore, obliged to discontinue it, and apply the felt splint as before.

May 1st. Is now entirely well. Amputated limb entirely healed, and the fractured arm perfectly strong and well set. He cannot flex the middle and ring fingers by themselves, but can do so by applying the under and ring fingers in close apposition to the former, and flexing them all together.

Another case is that of a white man, the sugar master, who received a sabre cut on the superior part of right parietal bone, extending over to the occipital bone. The wound was four inches long, and penetrated through both tables of the bone, leaving it, about the centre part of the dura mater, exposed to the eye; but not involving this membrane. Dressed with adhesive straps, and healed perfectly in two or three days without the patient's having ever had even slight fever.

[The late Sir Charles Bell, in his Institutes of Surgery, (vol. 1, p. 84,) speaking of Tetanus, says:—"What produces these terrible symptoms we hardly know. * * * * * Further we know nothing of the nature of this complaint." The important extensions, to our knowledge of the functions of the spinal system, made recently by Dr. Marshall Hall, and others, have led to the practical application of many of the leading physiological doctrines. In Tetanus the excitability of the whole spinal cord would appear to be morbidly increased, and a slight external stimulus produces reflex actions of a formidable character. In case second it will be found that any movement caused immediately violent spasms. In animals where this condition is artificially produced by strychnine, the same phenomena take place. Tetanus, no doubt, depends on an excited state of the whole excito-motory system, to which it is limited. The cerebral functions remain unimpaired until death.

In Traumatic Tetanus the seat of irritation is in the wound, and is proapgated to the nervous centres by the afferent nerves, and the irritation is then reflected throughout the muscular system by the efferent nerves, producing the terriffic convulsions which characterize the disease. It is worthy of remark that the muscles of the different orifices are primarily and chiefly affected, death being produced by spasm of the respiratory muscles. Obstinate constipation is a constant symptom, and frequently the contraction of the sphincter vesicæ is so strong as to induce complete retention of urine, as occurred in one of the above cases. In idiopathic Tetanus the origin of the disease is in the nervous centres, or true spinal cord itself. In the few autopsies we have of this disease, some change in the condition of the spinal cord and its membranes has been observed, but the exact lesion has not yet been accurately described. In the investigations, both physiological and pathological, of the nervous system which are now being instituted, may we not hope that attention will be directed to this fearful malady, and its kindred affection, hydrophobia, where the ganglia of the special senses and the cerebral apparatus would seem also to be involved. The treatment which hitherto has presented the best chance of success, has been founded on the pathological view here taken. Free depletion along the spine, followed by extensive violent counter irritation in the same region ; large doses of the narcotics, together with free purgation. In three cases out of five this treatment was successful in the hands of Dr. T. Harris, in the practice of the Pennsylvania Hospital. (See Medical Examiner, O. S., vol. 1, p. 323.)

